# The Effects of Money on Fake Rating Behavior in E-Commerce: Electrophysiological Time Course Evidence From Consumers

**DOI:** 10.3389/fnins.2018.00156

**Published:** 2018-03-19

**Authors:** Cuicui Wang, Yun Li, Xuan Luo, Qingguo Ma, Weizhong Fu, Huijian Fu

**Affiliations:** ^1^School of Management, Hefei University of Technology, Hefei, China; ^2^Key Laboratory of Process Optimization and Intelligent Decision-Making, Hefei University of Technology, Ministry of Education, Hefei, China; ^3^Academy of Neuroeconomics and Neuromanagement, Ningbo University, Ningbo, China; ^4^Business School, Ningbo University, Ningbo, China; ^5^Institute of Neural Management Sciences, Zhejiang University of Technology, Hangzhou, China; ^6^School of Management, Guangdong University of Technology, Guangzhou, China

**Keywords:** fake rating behavior, money, N2, LPP, neuromarketing

## Abstract

Online ratings impose significant effects on the behaviors of potential customers. Thus, online merchants try to adopt strategies that affect this rating behavior, and most of these strategies are connected to money, such as the strategies of returning cash coupons if a consumer gives a five-star rating (RI strategy, an acronym for “returning” and “if”) or returning cash coupons directly with no additional requirements (RN strategy, an acronym for “returning” and “no”). The current study explored whether a certain strategy (RN or RI) was more likely to give rise to false rating behaviors, as assessed by event-related potentials. A two-stimulus paradigm was used in this experiment. The first stimulus (S1) was the picture of a product with four Chinese characters that reflected the product quality (slightly defective vs. seriously defective vs. not defective), and the second stimulus (S2) displayed the coupon strategy (RN or RI). The participants were asked to decide whether or not to give a five-star rating. The behavioral results showed that the RI strategy led to a higher rate of five-star ratings than the RN strategy. For the electrophysiological time courses, the N1, N2, and LPP components were evaluated. The slightly defective products elicited a larger amplitude of the N1 component than the seriously defective and not-defective products, reflecting that perceptual difficulty was associated with the processing of the slightly defective products. The RI strategy evoked a less negative N2 and a more positive LPP than the RN strategy, indicating that the subjects perceived less conflict and experienced stronger incentives when processing the RI strategy. These findings will benefit future studies of fake online comments and provide evidence supporting the policy of forbidding the use of the RI strategy in e-commerce.

## Introduction

Online customer reviews are often thought of as electronic word of mouth (eWOM), which can help consumers simplify their search process and more easily determine product quality and fit uncertainty (Yin et al., 2014). A star rating (ranging from 1 to 5 stars) is typically included in each online review, and previous studies have noted that consumer-generated ratings have a substantial impact on the success or failure of a product in internet commerce (Chevalier and Mayzlin, 2006; Lafky, 2014). For example, it has been found that even one extra star in a Yelp review could increase revenues by 5–9% (Economist, 2015; Poddar et al., 2017). Given the great value of star ratings, online merchants have tried to adopt various marketing strategies to affect online rating behaviors, which may increase the number of false reviews.

Money has often been used as a tool in many marketing strategies, such as in the strategy of returning discount/cash coupons to consumers with no other request (RN strategy, RN is an acronym of “returning” and “no”). Some online sellers even return money or coupons directly if buyers give a five-star rating (RI strategy, RI is an acronym of “returning” and “if”), a practice that is ostensibly forbidden to be published on an online website by many e-commerce platforms (e.g., Taobao in China). However, this practice is still prevalent. For instance, the subjects in our experiment had received such incentives more than once for various mailed products. These strategies could result in false ratings. Only recently have researchers begun to analyze fraud in the context of online reviews (Hu et al., 2012; Poddar et al., 2017; Yamak et al., 2017). For example, Hu et al. (2012) proposed a statistical method for detecting false online review manipulation and assessed how consumers responded to products with manipulated reviews. Poddar et al. (2017) investigated the online rating bias that is elicited by false advertising and slander and mined big data to develop a method to measure online rating bias. These studies mainly used machine learning methods and focused on how to improve accuracy in identifying fake comments. However, from the perspective of consumer behavior, little is known about how the marketing strategies that are adopted by online merchants affect fake rating behavior in e-commerce and why online merchants adopt these illegal strategies.

The main distinction between the RI and RN strategies lies in the different forms that the monetary reward is given. Money, as a powerful social construct, can have a large impact on one's goals and behaviors. Several studies have demonstrated that money can increase the likelihood of self-interested or immoral behavior (Cullen et al., 1985; Agnew, 1994; Vohs et al., 2006; Vohs and Schooler, 2008; Kouchaki et al., 2013). In addition to the idea that money represents a reward in the feedback phase, mere exposure to money (as priming), devoid of any goal to which it might be relevant, could lead to behaviors that are relatively impersonal and self-focused (Vohs et al., 2006, 2008). The cash coupons that are used in the RI strategies are goal-related rewards that are given when the subjects performed the five-star rating behavior, whereas the cash coupons in the RN strategies are goal-unrelated rewards that are given to the subjects without any contingency. We suppose that the RI strategy decreases the evaluation process underlying rational choice and self-control and strengthens the motivation of consumers to engage in fake rating behaviors. However, there has not been direct behavioral or neurological evidence supporting this hypothesis.

To investigate how marketing strategies affect the fake rating behaviors that are observed in e-commerce in the context of an electrophysiological time course, event-related potentials (ERPs), a non-invasive brain scanning technique that measures the perceptual and cognitive processing of stimuli, were analyzed. Furthermore, the current study explored the moderating effect of product quality on the processing of different marketing strategies. This research furthers the study of online false reviewing and encourages e-commerce platforms, as well as government regulators, to realize the “darker side” of illegal online strategy manipulation.

Two ERP components have been associated with the processing of monetary rewards, namely, the N2 component and the late positive potential (LPP). N2 is a negative potential that peaks between 200 and 400 ms post-stimuli (Folstein and Van Petten, 2008; Dickter and Bartholow, 2010) and is consistently localized in the anterior cingulate cortex (ACC) (Nieuwenhuis et al., 2003; Yeung et al., 2004). There is evidence that the N2 component reflects conflict and mismatch from a visual modality (Van Veen and Carter, 2002; Folstein and Van Petten, 2008), which is sensitive to not only physical attribute conflicts but also perception conflicts (Ma et al., 2007, 2010; Han et al., 2015; Jin et al., 2017). For example, using a two-stimulus paradigm, Han et al. (2015) reported more negative N2 components when the second stimulus did not match the physical attributes of the first stimulus in terms of color or shape. In a brand extension evaluation task with pairs of stimuli, the N2 amplitude was found to be greater when the participant encountered a perceptual conflict between the brand name (S1) and the extension product name (S2) (Ma et al., 2007, 2010). In a study of immoral behaviors, Lahat et al. (2013) observed larger N2 amplitudes in response to moral violations than in response to conventional violations, and Yoder and Decety (2014) used a conflict-monitoring standpoint to explain the N2 component that is elicited by morally good and bad actions. Furthermore, researches have shown that compared with telling the truth, lying evokes greater N2 amplitudes (Wu et al., 2009; Suchotzki et al., 2015; Fu et al., 2017). The behaviors involved in giving a five-star rating to the defective products were immoral actions that were similar to deceptive behaviors, and subjects should detect a perceptual conflict, as reflected by the N2 component.

In addition to the N2 component, the LPP, as a later relevant component, occurs~300–800 ms post-stimulus. The LPP could be responsible for indexing later controlled processes that reflect cognitive reappraisal of stimuli, top-down cognitive control and attentional reallocation to motivationally salient stimuli (Sabatinelli et al., 2007; Dennis and Hajcak, 2009; Larson et al., 2009). In studies that have evaluated moral behavior, some researchers suggested that the LPP may be associated with conflict-resolution processing (Chiu Loke et al., 2011; Yoder and Decety, 2014; Wang et al., 2016), and moral actions were found to elicit greater LPP amplitudes than immoral actions (Yoder and Decety, 2014). Thus, the immoral action of giving a five-star rating to a defective product would evoke the LPP component. Moreover, in decision-making studies, the LPP (P3b) was found to be associated with the motivational significance of ongoing stimuli (Nieuwenhuis et al., 2005; San Martín, 2012), and the stimuli with stronger motivational impacts heightened the LPP amplitudes (Polezzi et al., 2010). The cash coupon incentives used in RI and RN strategies in exchange for providing a fake rating are different and, thus, might have different effects on the LPP component.

In the present experiment, we applied ERPs to investigate the neurophysiological processes of how the different monetary rewards used in the RI and RN strategies affect fake rating behaviors and to explore the moderating effect of product quality (slightly defective vs. seriously defective vs. not defective) on the processing of these strategies. The participants were asked whether they would give a five-star rating for different imperfect products using either the RI or RN marketing strategies. In the RI strategy, the participants would receive cash coupons only if they gave five stars to the defective products; whereas in the RN strategy, they would receive cash coupons without any additional contingencies. Thus we hypothesized that the goal-related monetary rewards used in RI strategy would alleviate the perceptual conflict of immoral action and had a stronger incentive than the goal-unrelated monetary rewards used in RN strategy, which were reflected by a less negative N2 amplitude and a larger LPP amplitude for RI in contrast to RN strategy, respectively. As a result, RI strategy might lead to a higher rate of giving five-star ratings. Meanwhile, product quality might have an impact on fake rating behavior such that a better product quality would result in a higher probability of giving five-star ratings, which might also be implicated by ERPs components. Overall, this study allowed us to explore how marketing strategies affect the immoral rating behaviors of customers at the neural level and to discover, from a customer's perspective, the reason why online sellers are willing to adopt illegal strategies.

## Materials and methods

### Participants

Twenty-one right-handed students from Ningbo University (10 females, all right-handed), aged 20–26 years (mean age = 23 ± 1.26 years), participated in this experiment. Information regarding the experiment was posted on the campus BBS (bulletin board system) to recruit the participants. All participants had experience in online shopping and were familiar with online marketing strategies (e.g., RI or RN strategies), were native Chinese speakers with no history of neurological or psychiatric abnormalities, and had normal or corrected-to-normal vision. The study was approved by the Internal Review Board of the Center for Management Decision and Neuroscience at Ningbo University. Before the experiment, written informed consent regarding issues such as awareness of the experimental task and protection of personal privacy, health, safety and dignity, was obtained from all the subjects in accordance with the Declaration of Helsinki. Participants were compensated for their time after the experiment. The electroencephalography (EEG) data of two participants were discarded due to excessive recording artifacts, leaving valid data for 19 participants (9 females and 10 males) for the final EEG data analysis.

### Experimental stimuli

A two-stimulus paradigm was used in the experiment. The first stimulus (S1) consisted of a product picture with a phrase below it that described the quality of the product. The product picture depicted either a sweater or a pair of shoes from the category of clothing to control for color, style and brand. Each phrase contained four Chinese characters, and the product quality included three categories: slightly defective (e.g., a small color difference), seriously defective (e.g., serious color difference) and not defective (e.g., no chromatic difference). Each of the quality categories comprised 5 phrases. Specifically, S1 consisted of 30 product pictures with phrase information reflecting the product quality. The second stimuli (S2) comprised 8 coupon strategies that were associated with the products chosen from two strategy categories (four coupons per category), namely, the strategy of returning cash coupons if given a five-star rating (RI strategy) and returning cash coupons with no contingencies (RN strategy). The stimuli used in the experiment consisted of 240 pairs of product pictures with product quality information (S1) and coupon strategies (S2), i.e., 2 pictures (sweater or shoes) × 3 categories of quality information × 5 phrases per quality category × 2 categories of coupon strategies × 4 coupons per strategy.

### Procedures

The participants sat in a comfortable chair to perform the experimental tasks in a sound-attenuated and electrically shielded room. A keypad was provided for the participants to input their choices. The stimuli were displayed in the center of a computer screen located 1 m away from the participant's eyes. The visual angle of all the stimuli was 4.58° × 4.58°. The E-prime 2.0 software package (Psychology Software Tools, Pittsburgh, PA, USA) was adopted to control the stimuli and acquire the behavioral data.

**Figure 1:**
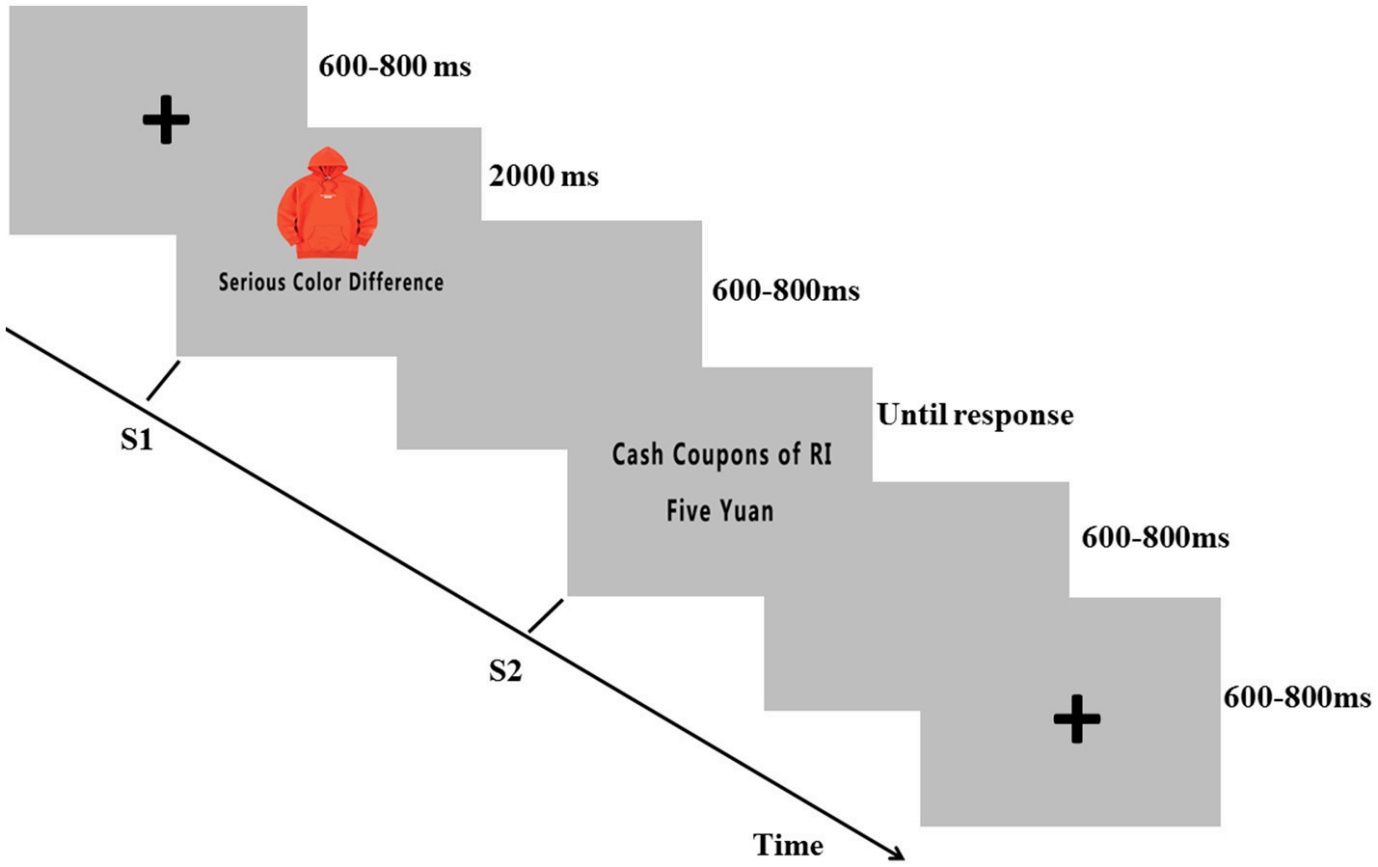
Experimental task: The participants were instructed to evaluate whether they would give a five-star rating for the S1 for different coupon strategies (RI or RN strategies) provided in S2.

The experiment consisted of four blocks, each containing 60 pairs of stimuli. For each trial of a block, first a fixation of “+” was presented against a gray background for a random interval from 600 to 800 ms; then, S1 was presented for 2,000 ms followed by a blank screen ranging from 600 to 800 ms between S1 and S2. S2 disappeared until a response was made, followed by presentation of a blank screen for 600 to 800 ms (as shown in Figure 1). The participant was able to rest for several minutes after each block.

The participants were first provided the following introduction scenario: “You received a sweater or a pair of shoes from taobao.com, and you checked the quality of the product (the quality result was reflected by S1). Additionally, you found a cash coupon associated with the product given by the online sellers, which was reflected by S2. Please evaluate whether you would give a five-star rating according to the information of S1 and S2 by using the keypad.” After the introduction, each subject performed 10 training trials to become familiarized with the procedure. The response-to-hand assignments were counterbalanced across all the subjects. The participants were paid 50 Chinese yuan (approximately US$7) as payment.

### ERP recording and analysis

In this experiment, EEG data were recorded (sampling rate of 1,000 Hz) using a NeuroScan SynAmps2 Amplifier (Curry 7, Neurosoft Labs, Inc., Virginia, USA) and a cap containing 64 Ag/AgCl electrodes. The cephalic (forehead) location served as the ground, and the left mastoid served as an online reference. The vertical and horizontal electrooculograms (EOGs) were recorded with a pair of electrodes placed above and below the left eye (vertical EOG), and another pair of electrodes placed 10 mm from the lateral canthi of both eyes (horizontal EOG). The electrode impedance was maintained below 5 kΩ during the experiment.

### Electroencephalogram analysis

The NeuroScan analysis software (Scan 4.5, Neurosoft Labs, Inc., Virginia, USA) was used to process the offline EEG signals. The EEG signals were digitally filtered with a low-pass filter at 30 Hz (24 dB/Octave). Any EOG artifacts were corrected by the method proposed by Semlitsch et al. (1986) for all subjects. The EEG recordings were segmented into epochs from 200 ms before the onset of the second stimulus (S2) to 800 ms after the onset of S2, with the prestimulus period used as baseline. Any trials with electro-oculography activity or other artifacts (such as amplifier clipping, bursts of electromyographic activity or peak-to-peak deflections exceeding ±100 μV) were excluded, and more than 30 sweeps for each condition remained. The EEG recordings for each participant were averaged separately within the six conditions (3 categories of product quality× 2 categories of coupon strategies).

Based on visual inspection of the grand average waveforms and the related studies mentioned in the introduction, the N1, N2, and LPP components were analyzed in our experiment. To analyze the mean amplitudes of the N1, N2, and LPP components, the time window for the N1 component was specified as 100–120 ms after the onset of S2, the N2 component as 270–370 ms, and the LPP component as 400 ms to 600 ms. According to the brain locations of the ERP components and the guidelines given by Gui et al. (2016), the nine electrodes corresponding to the coronal and sagittal factors, i.e., the F3, FZ, F4, FC3, FCz, FC4, C3, Cz, and C4 electrodes in the frontal, fronto-central and central areas, were used for N1 and N2, and the C3, Cz, C4, CP3, CPz, CP4, P3, Pz, and P4 electrodes in the central, centro-parietal and parietal areas were used for LPP. Repeated measures analyses of variance (ANOVAs) were conducted using SPSS (SPSS 16.0, SPSS, Inc., Chicago, IL) separately for the N1, N2, and LPP components. The within-subject factors consisted of the 3 categories of product quality (slightly defective vs. seriously defective vs. not defective), the 2 categories of coupon strategy (RI strategy vs. RN strategy), and the 9 electrodes. The Greenhouse–Geisser correction was used when necessary (uncorrected *df* is reported with the ε and corrected *p*-values), and the Bonferroni correction was used for multiple paired comparisons.

## Results

### Behavioral results

**Figure 2:**
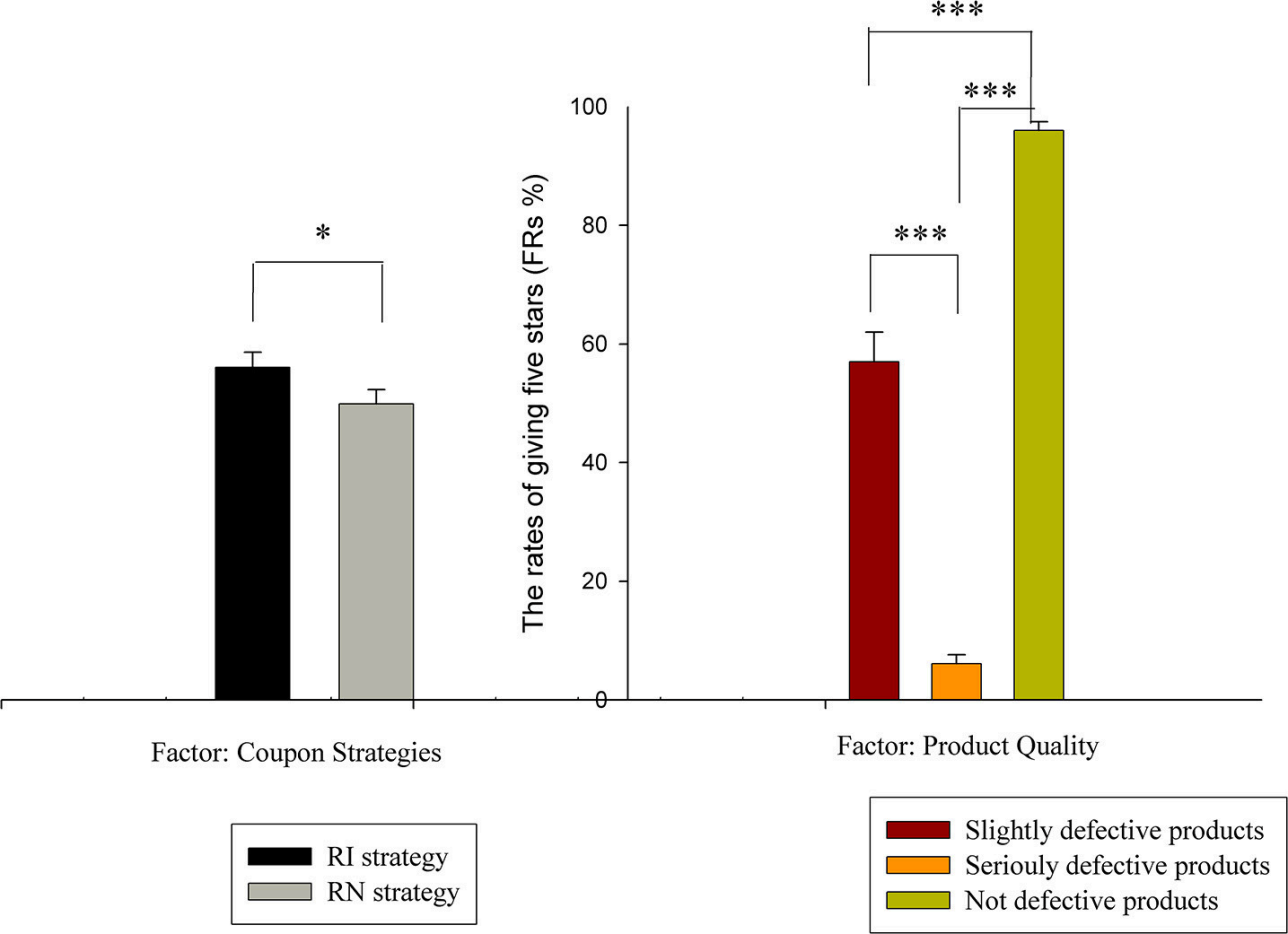
FR results. The rates of giving five-stars for the factors of coupon strategy (RI vs. RN) and product quality (slightly defective products vs. seriously defective products vs. not defective products). ^*^p < 0.05, ^***^p < 0.001.

The rates that participants gave five-star ratings (FRs) and their reaction times (RTs) were analyzed separately by within-subject ANOVAs with factors of product quality (3 categories: slightly defective vs. seriously defective vs. not defective) and coupon strategy (2 categories: RI strategy vs. RN strategy). Regarding the FRs, there were significant main effects of the coupon strategy [*F*_(1, 20)_ = 6.278, *p* < 0.05, η^2^ = 0.239] and product quality [*F*_(2, 40)_ = 245.873, ε = 0.690*, p* < 0.001, η^2^ = 0.925] factors, with no interaction effect between the two factors. The FR of the RI strategy (*M* = 0.561, *S.E*. = 0.025) was higher than that of the RN strategy (*M* = 0.499, *S.E*. = 0.024). For the factor of the product quality, Bonferroni-corrected pairwise comparisons showed that the FR of the not defective products (*M* = 0.960, *S.E*. = 0.015) was larger than that of both the slightly defective products (*M* = 0.570, *S.E*. = 0.050) (*p* < 0.001) and the seriously defective products (*M* = 0.061, *S.E*. = 0.015) (*p* < 0.001). Furthermore, the FR of the slightly defective products was larger than that of the seriously defective products (*p* < 0.001). The results of the FRs are shown in Figure 2.

**Figure 3:**
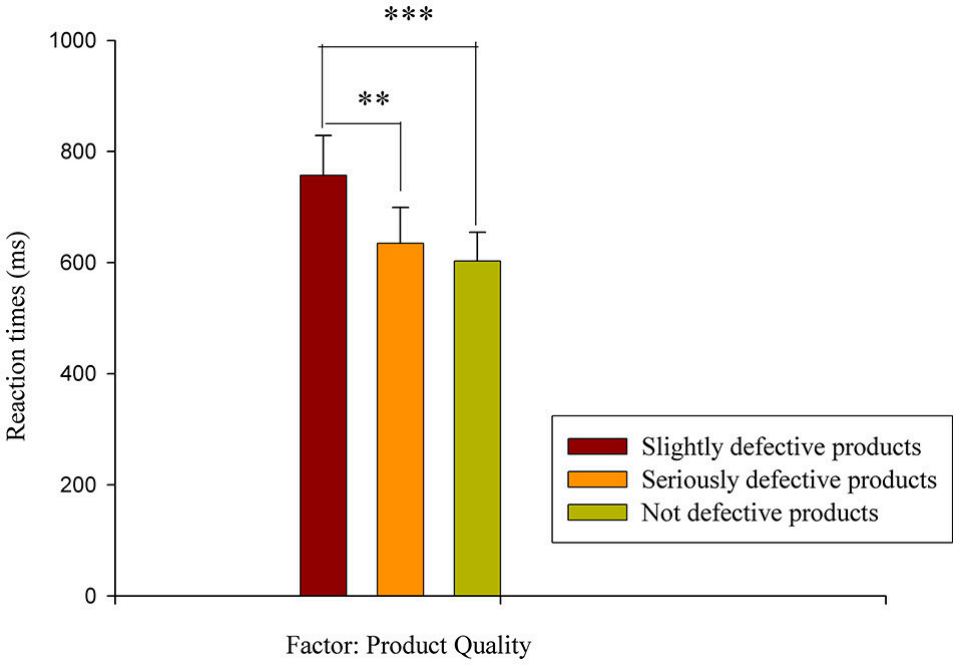
RT results. The reaction times for the product quality factor (slightly defective products vs. seriously defective products vs. not defective products). ^**^p < 0.01, ^***^p < 0.001.

Regarding the RTs, the two-way 3 (product quality) × 2 (coupon strategy) within-subjects ANOVA showed a significant main effect of the product quality factor [*F*_(2, 40)_ = 13.834, *p* < 0.001, η^2^ = 0.409], with no salient main effect of the coupon strategy factor and no interaction effect between product quality and coupon strategy. The pairwise comparison test for the product quality factor showed that the RT for the slightly defective products (*M* = 757.381, *S.E*. = 71.856) was longer than the RTs of the seriously defective products (*M* = 634.908, *S.E*. = 64.777) (*p* < 0.001) and the not defective products (*M* = 603.061, *S.E*. = 51.566) (*p* < 0.01). However, no significant difference in RT was observed between the seriously defective products and the not defective products (*p* > 0.05) (as shown in Figure 3).

In addition, to explore if there were gender differences with regard to the behavioral results, mixed-design ANOVAs were performed separately on FRs and RTs, including gender as a between-subject factor. However, neither the main effects of gender nor any interactions involving gender were significant (*ps* > 0.05).

### EEG results

**Figure 4:**
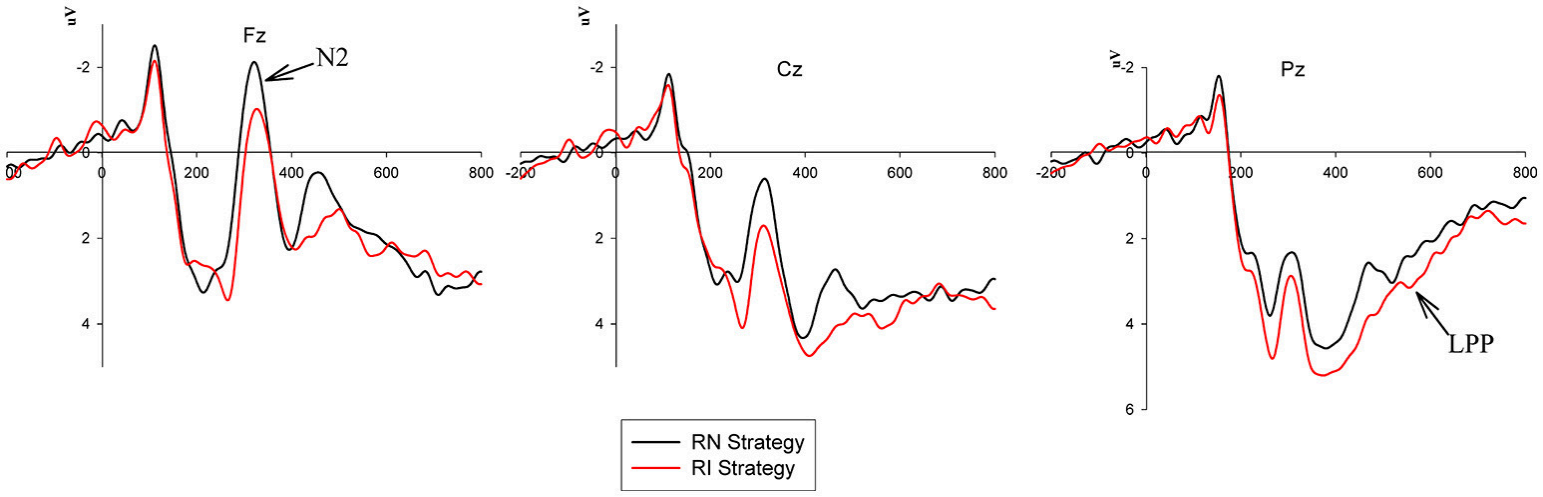
Grand-average ERP waveforms of the frontal, central and parietal regions collected from the Fz, Cz, and Pz electrodes. Comparison of the amplitudes of the N2 and LPP components between the two conditions: the RI strategy vs. the RN strategy.

**Figure 5:**
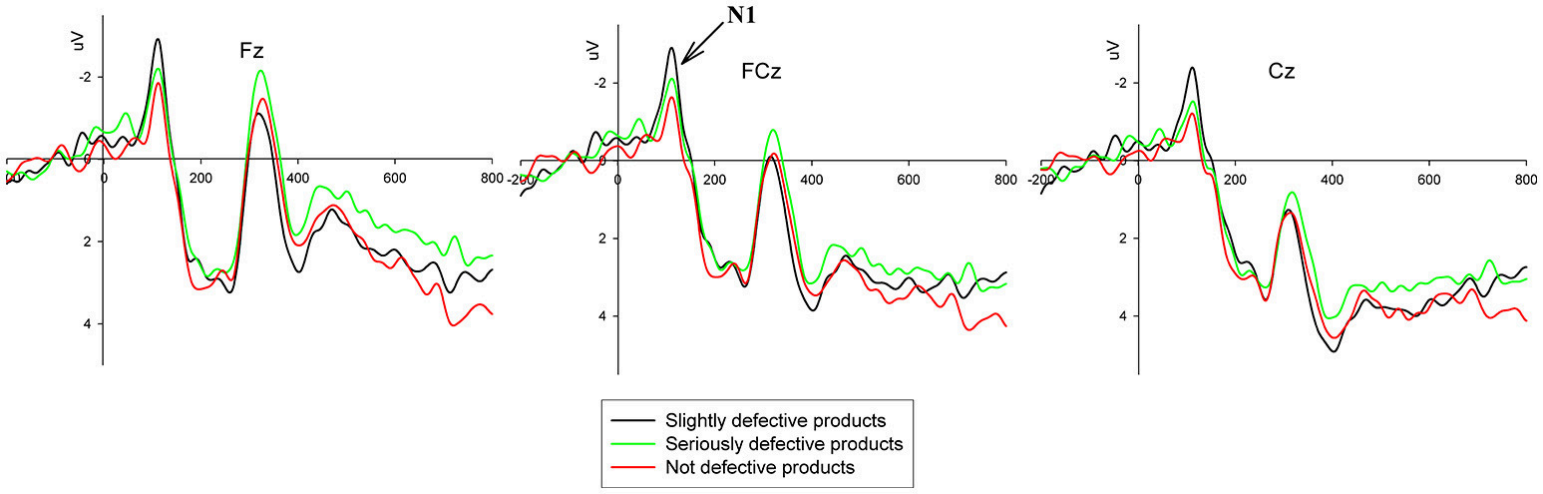
Grand-average ERP waveforms of the frontal, fronto-central and central regions recorded from the Fz, FCz, and Cz electrodes. N1 amplitude comparison of the three product conditions, i.e., slightly defective products vs. seriously defective products vs. not defective products.

The grand-average ERPs for the factors of the coupon strategy and product quality are shown in Figures 4, 5, respectively.

A three-way 3 (quality phrase: slightly defective vs. seriously defective vs. not defective) × 2 (coupon strategy: RI strategy vs. RN strategy) × 9 (electrode) within-subjects ANOVA for N1 in the time window from 100 to 120 ms was conducted. There was a significant main effect of the product quality factor [*F*_(2, 36)_ = 4.968, *p* < 0.05, η^2^ = 0.216], with no significant effect of the coupon strategy and no interaction effect between the product quality and coupon strategy factors. Bonferroni-corrected pairwise comparisons showed that the average N1 amplitude in the condition of a slightly defective product (*M* = −2.420, *S.E*. = 0.353) was marginally more negative than that in the condition of a seriously defective product (*M* = −1.744, *S.E*. = 0.434) (*p* < 0.1) and was significantly more negative than that in the condition of a not defective product (*M* = −1.412, *S.E*. = 0.525) (*p* < 0.01), with no salient difference between the seriously defective product and not defective product conditions (*p* > 0.1).

An ANOVA for the mean N2 amplitude in the 270–370 ms time window was also conducted and revealed a significant effect of the coupon strategy factor [*F*_(2, 36)_ = 6.132, *p* < 0.05, η^2^ = 0.254], and the N2 amplitude elicited by the RN strategy (*M* = 0.558, *S.E*. = 0.669) was more negative than that elicited by the RI strategy (*M* = 1.378, *S.E*. = 0.605). There was no salient effect of the product quality factor and no interaction effect between the product quality and coupon strategy factors.

An ANOVA for the mean LPP amplitude in the 400–600 ms time window revealed a significant main effect of the coupon strategy factor [*F*_(2, 36)_ = 4.914, *p* < 0.05, η^2^ = 0.214]. The RI strategy (*M* = 3.035, *S.E*. = 0.552) evoked a larger LPP component than the RN strategy (*M* = 3.035, *S.E*. = 0.552). There was no significant main effect of the product quality factor, and furthermore, no interaction effect was found between the product quality and coupon strategy factors.

To investigate if there were gender differences in the EEG results, mixed-design ANOVAs that included gender as a between-subject factor were performed separately for the N1, N2, and LPP amplitudes. However, in line with the behavioral results, there were no main effects of gender or any interaction effects involving gender for any of the ERP components (*ps* > 0.05).

## Discussion

Researchers have recently expressed increasing interest in employing neuroscientific tools to investigate the neural basis of marketing phenomena. For example, several studies have used ERPs to explore the neural basis of brand extension in order to determine a strategy to enhance the success of brand extension (Ma et al., 2007, 2010; Shang et al., 2017). In the present study, we investigated both how coupon strategies affect the fake rating behaviors of online customers and the temporal dynamics of the neural activity that are associated with different marketing strategies. These findings helped us to further understand why online platforms forbid certain strategies, such as money being returned if a customer provides a five-star rating, at the neurological level. Additionally, the moderating effect of the product quality received from online shopping was considered in this study.

Behaviorally, a remarkable FR effect was found based on the coupon strategy (i.e., customers were more willing to give a five-star rating with the RI strategy compared to the RN strategy). According to the Tool Theory of money motivation, monetary incentives have effects on behavioral performance, which result not only from the monetary rewards in the feedback stage but also from thinking about money, even unconsciously through priming (Lea and Webley, 2006; Vohs et al., 2006; Zhou et al., 2009; Ma et al., 2015). However, different forms of giving monetary rewards were found to have different levels of attractiveness. In our experiment, the RI strategy had a stronger effect on rating behaviors than the RN strategy. That is, compared with giving cash coupons without any contingencies, people tended to give more five-star ratings when the decision led to a monetary reward. Thus, the strategy that involved returning cash coupons as monetary rewards if a five-star rating was given increased the falsity of the review comments to a greater extent than other strategies. Additionally, the FR was inversely related to the quality of the products received from the online platform. That is, products with higher quality are more likely to receive a five-star rating by consumers. However, the product quality had no moderating effect on the validity of the coupon strategy. Thus, regardless of the product quality, the RI strategy would induce greater falsity of the rating comments than the RN strategy.

In terms of RTs, a significant effect was found for the product quality factor; slightly defective products corresponded to longer RTs than not defective and seriously defective products, with no difference in RT between the latter two. Previous studies have found that the task difficulty affects RT, with more difficult tasks requiring more time to process (Ma et al., 2014; Dunn et al., 2017). It was relatively easier for the participants to decide whether to give a five-star rating in the not defective product and seriously defective product conditions. In terms of task difficulty, the slightly defective product condition required more mental resources to complete the rating task, which led to longer RTs. However, there was no significant difference between the RI strategy and the RN strategy, and there was no interaction effect between the product quality and the coupon strategy.

At the brain level, three components (N1, N2, and LPP) were identified in this study. N1, as an early ERP component, can reflect early automatic perceptual processes; this component has been found to be affected by attentional factors (e.g., Luck et al., 1994) and perceptual difficulty (Handy and Mangun, 2000). We found a significant main effect of product quality on the amplitude of the N1 component. The slightly defective products elicited higher N1 amplitudes than the seriously defective products and the not defective products, with no difference in N1 amplitude between the latter two. These results indicated that the participants perceived the perceptual difficulty associated with the slightly defective products at an early stage and paid more attention to processing them. Combined with the results of the RTs, we speculated that the decision of whether to give a five-star rating for the slightly defective products was more difficult than the other decisions. The difficulty of this decision could be automatically perceived during an early stage at the brain level and led to longer RTs at the behavioral level. However, the different coupon strategies did not evoke differences in the N1 amplitude, indicating that the coupon strategy factor was not deeply processed at an early stage.

The N2 component, as aforementioned in the introduction section, could reflect the process of detecting conflicting information (Van Veen and Carter, 2002; Ma et al., 2007, 2010; Folstein and Van Petten, 2008; Lahat et al., 2013), for which stronger cognitive conflicts induce larger N2 amplitudes (Fu et al., 2017). Monetary incentives make participants less sensitive to conflict or distress by communal values, which increases the likelihood of self-interested or immoral behavior (Cullen et al., 1985; Agnew, 1994; Vohs et al., 2006, 2008; Kouchaki et al., 2013). Thus, different forms of monetary access could reduce the conflicted perception of immoral behaviors to different degrees, which can be reflected by the amplitude deflection of the N2 component. In the current study, the RI strategy elicited a less negative N2 component than the RN strategy, which indicated that the participants detected less conflict in giving a five-star rating in response to the RI strategy than in response to the RN strategy. More specifically, the goal-related monetary rewards obtained from the RI strategy decreased the cognitive conflict compared to the goal-unrelated monetary rewards obtained from the RN strategy, as the former strategy made participants more willing to give five-star ratings to gain the reward.

Moreover, an obvious LPP component was observed from 400-600 ms. The LPP component has been reported to be associated with the conflict-resolution processing of stimuli evaluation (Chiu Loke et al., 2011; Yoder and Decety, 2014; Wang et al., 2016) and to be sensitive to the motivational significance of stimuli (Nieuwenhuis et al., 2003; San Martín, 2012). In the current study, after detecting the conflict, as reflected by the N2 component, a controlled and elaborate process was deployed for conflict resolution, which was reflected by the LPP component. The LPP amplitude that was evoked by the RI strategy was larger than that evoked by the RN strategy, which showed that the RI strategy had a greater incentive effect for the subjects to resolve the conflict than the RN strategy. Though the same amount of cash coupons were returned to the consumers, setting a related goal evoked a stronger motivation than not doing so. This incentive effect still existed even if the action required to achieve the goal was immoral.

The N1, N2, and LPP ERP components in the current study, may reflect the three-stage process involving how coupon strategy and quality information affect the fake rating behavior of consumers in e-commerce. The first stage was automatic sensory processing, which was reflected by the N1 component. The subjects automatically perceived the difficulty of the different levels, which was affected by the product quality information with no significant processing of the coupon strategy; the second stage involved the processing required for conflict detection, which was reflected by the N2 component. According to the different coupon strategies, the subjects detected different levels of cognitive conflict in the task of giving five-star ratings to the products with different qualities. The third stage involved motivational and conflict-resolution processing, as reflected by the LPP component, and the different coupon strategies were controllably analyzed and evaluated.

The three-stage process related to the fake rating behaviors resulting from different marketing strategies exhibited some similarities to the neural processes involved in moral decision-making, which include automatic processes (N1), emotional perception processes (N2) and controlled and elaborative processes (LPP) (Yoder and Decety, 2014; Gui et al., 2016). As mentioned in the introduction section, money can increase the likelihood of self-focused or immoral behavior (Cullen et al., 1985; Agnew, 1994; Vohs et al., 2006; Vohs and Schooler, 2008; Kouchaki et al., 2013). The cash coupons in the current study were specific monetary rewards, meaning that they could also lead to relatively self-interested rating behaviors. The fake five-star rating behaviors induced by the cash coupons have great impacts on other consumers' attitudes and purchase decisions. Thus, giving a fake rating could be considered an immoral behavior to some extent.

With the growing popularity of online shopping, fake online review has drawn increasing scholarly attention. The present study was undertaken from the perspective of consumers and used ERP measures to investigate if monetary reward offered by online sellers could give rise to consumers' fake rating behavior and how. We conjecture that the ERP findings of the current study might to a certain extent reflect the general tendencies of the neurocognitive processes underlying fake rating behavior. A fake rating is a rating not in line with the truth. Thus, giving a fake rating is similar to deception, which in many cases is a type of immoral behavior and may result in greater cognitive conflict than giving a truthful rating, which could be reflected by the N2 component (Wu et al., 2009; Suchotzki et al., 2015; Fu et al., 2017). Moreover, a related goal for the consumers, such as earning a certain amount of monetary reward and maintaining good interpersonal relationship, could not only alleviate the perceptual conflict but also prompt them to have greater incentives to give fake high-score ratings, which would be indicated by a smaller N2 and a larger LPP amplitude. Consequently, the illegal strategies used for manipulating fake rating behavior, particularly those capable of reducing cognitive conflict and strengthening incentives, should be strictly prohibited.

## Conclusions

In this study, we used ERPs to explore which marketing strategy (RI vs. RN strategy) more strongly affects the online fake rating behavior of consumers. The RI strategy increased the rate of the five-star rating behavior compared with the RN strategy, with no observed moderating effect of the product quality. At the level of the brain, the N1, N2 and LPP components were found to reflect the neurophysiological processes involved in the task. The processing of the slightly defective products was perceived to be more difficult than the processing of the seriously defective and not defective products, as reflected by the N1 component. In addition, less conflict and stronger incentives were detected during the RI strategy than the RN strategy, as reflected by the N2 and LPP components, respectively. Generally, the goal-related monetary rewards involved in the RI strategy enhanced the falsity of the online comments by both reducing the perception of conflict and increasing the motivation, and these influences warrant additional future studies of fake online comments. To the best of our knowledge, the current study is among the first to explore the effects of money on fake rating behavior and the associated neural correlates. These findings could facilitate the study of online false reviewing and help platforms or government regulators uncover the possible harms from illegal online strategy manipulation.

## Author contributions

CW, HF, and QM conceived and designed the experiments. CW, YL, and QM performed the experiments. CW, YL, and XL analyzed the data. CW, XL, and HF wrote and refined the article. CW, WF, and HF participated in the revision of the article.

### Conflict of interest statement

The authors declare that the research was conducted in the absence of any commercial or financial relationships that could be construed as a potential conflict of interest.
