# Corrigendum: The Effects of Money on Fake Rating Behavior in E-Commerce: Electrophysiological Time Course Evidence From Consumers

**DOI:** 10.3389/fnins.2019.00192

**Published:** 2019-03-11

**Authors:** Cuicui Wang, Yun Li, Xuan Luo, Qingguo Ma, Weizhong Fu, Huijian Fu

**Affiliations:** ^1^School of Management, Hefei University of Technology, Hefei, China; ^2^Key Laboratory of Process Optimization and Intelligent Decision-Making, Hefei University of Technology, Ministry of Education, Hefei, China; ^3^Academy of Neuroeconomics and Neuromanagement, Ningbo University, Ningbo, China; ^4^Business School, Ningbo University, Ningbo, China; ^5^Institute of Neural Management Sciences, Zhejiang University of Technology, Hangzhou, China; ^6^School of Management, Guangdong University of Technology, Guangzhou, China

**Keywords:** fake rating behavior, money, N2, LPP, neuromarketing

There was an error in the **Acknowledgments**. The correct funding number for the “National Project” funder is “AWS14J011.”

The authors apologize for this error and state that this does not change the scientific conclusions of the article in any way. The original article has been updated.

